# Exploring Salivary Epithelial Dysfunction in Sjögren’s Disease

**DOI:** 10.3390/ijms25094973

**Published:** 2024-05-02

**Authors:** Braxton Noll, Micaela Beckman, Farah Bahrani Mougeot, Jean-Luc Mougeot

**Affiliations:** 1 Translational Research Laboratories, Cannon Research Center and Department of Oral Medicine, Oral and Maxillofacial Surgery, Atrium Health Carolinas Medical Center, 1542 Garden Terrace, Charlotte, NC 28203, USA; 2 Department of Otolaryngology, Wake Forest University School of Medicine, 475 Vine Street, Winston-Salem, NC 27101, USA

**Keywords:** Sjögren’s disease, extracellular matrix, salivary gland hypofunction, epithelial–mesenchymal transition

## Abstract

Sjögren’s Disease (SjD) is an autoimmune disease of the exocrine tissues. Etiological events result in the loss of epithelial homeostasis alongside extracellular matrix (ECM) destruction within the salivary and lacrimal glands, followed by immune cell infiltration. In this review, we have assessed the current understanding of epithelial–mesenchymal transition (EMT)-associated changes within the salivary epithelium potentially involved in salivary dysfunction and SjD pathogenesis. We performed a PubMed literature review pertaining to the determination of pathogenic events that lead to EMT-related epithelial dysfunction and signaling in SjD. Molecular patterns of epithelial dysfunction in SjD salivary glands share commonalities with EMT mediating wound healing. Pathological changes altering salivary gland integrity and function may precede direct immune involvement while perpetuating MMP9-mediated ECM destruction, inflammatory mediator expression, and eventual immune cell infiltration. Dysregulation of EMT-associated factors is present in the salivary epithelium of SjD and may be significant in initiating and perpetuating the disease. In this review, we further highlight the gap regarding mechanisms that drive epithelial dysfunction in salivary glands in the early or subclinical pre-lymphocytic infiltration stages of SjD.

## 1. Epidemiology and Pathology of Sjögren’s Disease

Sjögren’s Disease [SjD] is a chronic autoimmune disease affecting 0.01–4.8% of the general population with a 9:1 female-to-male ratio [1,2]. There is no cure for SjD, and current therapies mainly target symptoms [3]. Salivary hypofunction is associated with lymphocytic infiltration of glandular tissue and other factors altering acini structural integrity [4]. Arthralgia, fatigue, myalgia, and Raynaud’s phenomenon affect 75% of SjD patients [5]. SjD-associated systemic complications include nephritis, skin vasculitis, and peripheral neuropathy [6]. There is an increased risk for non-Hodgkin’s B-cell lymphoma [NHL] in SjD patients who have a 16-fold higher likelihood of developing NHL, with a cumulative risk of 9.8%, fifteen years following SjD clinical onset [7,8]. In SjD patients, NHL development mostly involves mucosa-associated lymphoid tissue [MALT] [9]. Moreover, about 60% of patients have another autoimmune disease, such as systemic lupus erythematosus [SLE] and rheumatoid arthritis [RA] [10]. Twenty percent of RA patients are diagnosed with secondary Sjögren’s disease [sSjD] [6]. Up to 46% of SLE patients and 21% of RA patients report reduced tear production, while 26% and 11% report a subjective sensation of dry eyes (xerophthalmia), respectively [11]. 

In the absence of anti-SSA autoantibody serum positivity, SjD classification involves the determination of the extent of immune cell infiltration in a minor labial salivary gland [MLSG] biopsy [12,13,14]. Infiltration is determined by the number of foci (cell aggregates) present, with each consisting of ≥50 immune cells within a 4 mm^2^ area of glandular tissue. The number of aggregates corresponds to a focus score [FS] ranging from 0 to 12 on an ordinal scale, with a score of 12 representing foci confluency [2,12,15,16,17,18,19]. However, 15% of patients with FS 2–6 do not report dry mouth/eyes [20]. Patients with non-SjD sicca complex (i.e., *sicca* syndrome patients) also have dry mouth symptoms. The terminology of ‘*sicca*’ aligns with the concept of xerostomia, which refers to the subjective complaint of mouth dryness possibly associated with a change in saliva composition/quality and not necessarily with hyposalivation causing a reduction in saliva production, i.e., an objective sign of dry mouth [21]. Non-autoimmune xerostomia may be caused by radiation therapy during head and neck cancer treatment or by the routine intake of multiple medications [22]. 

There are categories of patients with xerostomia that can be distinguished from *sicca* patients [6]. Here, we define *sicca* patients as xerostomic patients who do not meet SjD diagnostic criteria per the 2016 American College of Rheumatology—European League Against Rheumatism [ACR-EULAR] classification guidelines [15]. Also, *sicca* patients may experience dry mouth/eyes without substantial immune cell infiltration and in the absence of detectable levels of serum anti-Sjögren’s syndrome-related antigen A [anti-SSA] autoantibodies and FS < 1 [15]. The salivary glands of *sicca* patients may exhibit both histopathological signs of chronic inflammation, such as fibrosis with limited immune cell infiltration, and some expression of SjD pathogenesis markers. 

**Figure 1:**
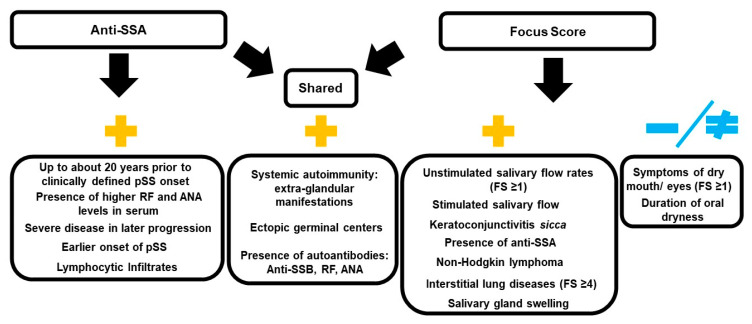
Associations between diagnostic markers anti-SSA and/or focus score [FS] and their relationship to epithelial disease progression and activity in SjD. Legend: Relationships regarding the presence of anti-SSA and/or focus score [FS] with symptoms and manifestations of SjD are shown. Features positively associated with both anti-SSA and FS diagnostic markers are listed under the shared header. Positively associated symptoms/manifestations represented under plus signs (+) are listed under their respective diagnostic feature (anti-SSA or FS). Symptoms/manifestations not clearly associated with FS are listed under the blue (−/≠) symbol. Overall, diagnostic markers (anti-SSA and FS) overlap or represent patients sharing a large degree of clinical manifestations. However, neither criterium provides an adequate measure or association with glandular function or atrophy. RF is rheumatoid factor; ANA is antinuclear autoantibody. References: Abd-Allah NM et al., 2019; Carrubi F et al., 2015; Daniels TE et al., 2011; Fayyaz et al., 2016; Kakugawa T et al., 2018; Leehan KM et al., 2018; Radfar L et al. 2002; Risselada AP et al. 2013, 2014; Theander E et al. 2015; Wei P et al. 2015; Wise CM and Woodruff RD, 1993.

Overall, SjD classification does not necessarily account for non-immune-driven glandular dysfunction and early disruption of acini integrity [6]. In addition, a fraction of anti-SSA-negative *sicca* patients eventually become anti-SSA-positive as the disease progresses [23]. Early anti-SSA presence in serum may define a subset(s) of SjD patients [24,25]. Different etiologies and pathophysiological mechanisms are involved during SjD progression, leading to salivary gland destruction [24]. Autoimmunity is a widely accepted hallmark of SjD per the ACR-EULAR 2016 classification criteria [15]. However, the role of the immune system in SjD is not fully elucidated because the salivary epithelium itself can generate activation signals leading to immune cell infiltration [26]. Thus, relationships between classification criteria such as FS, the presence of serum anti-SSA autoantibodies, epithelial dysfunction, and disease activity established with the EULAR Sjögren’s syndrome disease activity index [ESSDAI] are complex [27]. This highlights the need to develop novel biomarker-based approaches for early interventions to reduce or prevent the systemic progression of the disease (Figure 1).

## 2. Methodology and Study Design

We conducted a review of the literature using PubMed, which did not involve a formal comparative meta-analysis. A conventional literature search using PubMed was conducted on all studies using the search terms “Sjögren’s Disease” or “Sjögren’s Syndrome” and “Extracellular Matrix” [ECM] or “ECM” or “Epithelial–Mesenchymal Transition” or “EMT”. Returned abstracts were used to filter relevant studies based on keywords presented in Supplemental File S1. Articles related to the topic of epithelial dysfunction in the process of SjD development and the peripheral pathological processes accompanying or driving EMT changes were retained for review. A Preferred Reporting Items for Systematic Reviews and Meta-Analyses [PRISMA] table of criteria to assess bias is provided in Supplemental File S2. Justifications for bias are provided in Supplemental File S3a,b for all cited references and PubMed Identification [PMID] citations in Figure 1 and Table 1, respectively.

## 3. Significance of the Salivary Epithelium in SjD Development and Challenges in Targeting the Pre-Symptomatic Phase

### 3.1. SjD Pathogenic Processes during Subclinical/Pre-Symptomatic and Clinical Phases

**Figure 2:**
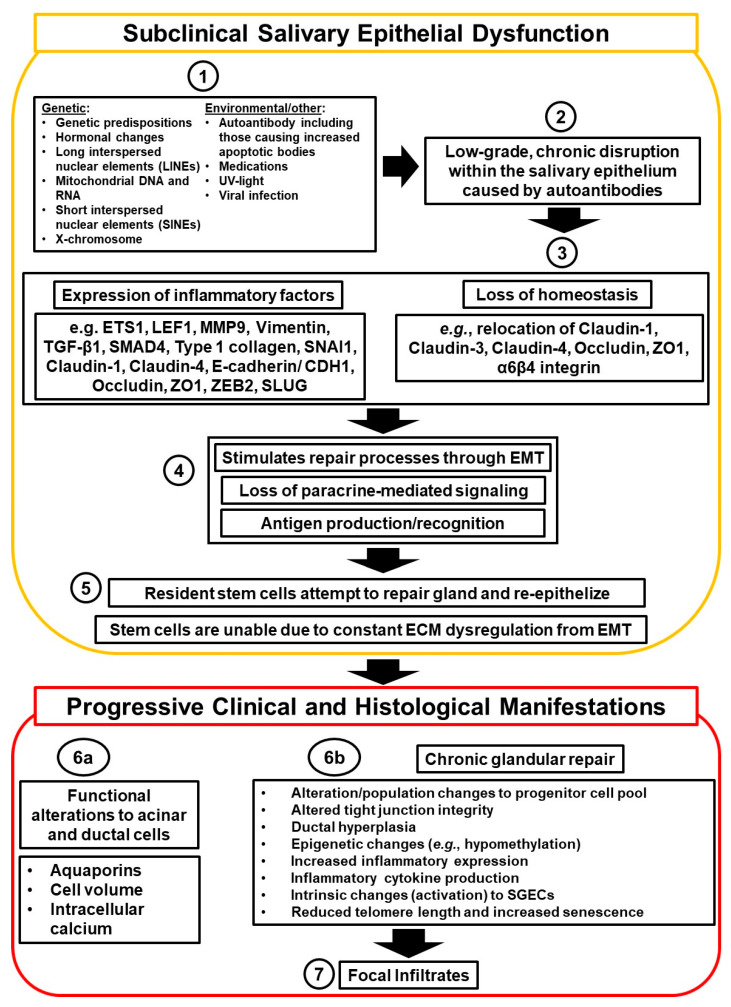
Proposed model for salivary epithelium-driven pathogenesis of SjD in subclinical salivary epithelial dysfunction and clinical histological manifestation phases.

A proposed model pertaining to subclinical salivary epithelial dysfunction and clinical histological manifestation stages of epithelium-driven SjD development is presented in Figure 2. Several etiological factors are implicated in SjD pathogenesis, including hormonal changes, viral infection, genetic predisposition, immune system defects, and gender (X-chromosome) (Figure 2, Section 1) [28,29]. Since the onset of symptoms can occur several years before clinically defined SjD onset, the effects of the etiological factor could be interpreted as a low-grade, chronic epithelial and immunological disruption (Figure 2, Section 2) [30]. The disruption of salivary gland epithelium homeostasis may reach a ‘critical mass’ towards further disease progression (Figure 2, Section 3) [26]. This disruption reflects de-differentiation of acinar cells after explant culture or when human parotid gland cells are exposed to various inflammatory cytokines (Figure 2, Section 3) [31]. In early SjD, the activity and properties of the salivary gland epithelial cells (SGECs) change, shifting towards a damaged-repair/wound-healing phenotype as seen in epithelial–mesenchymal transition [EMT] (Figure 2, Section 4) [32]. The change in acinar paracrine function was shown to result from explant culture stress through Src and p38-MAPK pathways, although the exact “stressor” remains unknown (Figure 2, Section 4) [31]. 

Legend.

1. The pathogenesis of SjD arises from a complex etiology where several possible predisposing factors have been linked to its development.
2. Combinations of genetic and environmental perturbations eventually lead to loss of salivary epithelium integrity and, hence, acini structures.
3. Homeostatic disruption by etiological factors promotes local inflammatory processes that are associated with repair-signaling pathways.
4. Repair of the glandular epithelium is mediated through epithelial–mesenchymal transition (EMT), where acinar and ductal cells lose their epithelial characteristics and take on a mesenchymal-like phenotype. It is unclear at which stages increased antigen expression or recognition by immune cells occurs predominantly.
5. Extracellular matrix (ECM) is damaged through enhanced expression and/or activity of proteases, including matrix metalloproteinase-9 (MMP9), providing an inadequate foundation for the re-epithelization by resident stem/progenitor cells relying on signaling molecules from the ECM. Resident stem cells/progenitor cells are unable to re-epithelize due to constant ECM dysregulation from EMT.
6. Disorganization and disrepair of the salivary epithelium are mirrored by the acinar and ductal cell populations, exhibiting changes to aquaporin localization and/or expression, altered cell volume, and improper intracellular calcium signaling (6a), resulting in functional alterations of the secretory cells involved in chronic glandular repair (6b).
7. Eventually, mechanisms dictating the chronic state of dysfunctional repair within the salivary epithelium contribute to SjD pathogenesis and lead to substantial infiltration by immune cells into the glandular tissue.

In SjD, epithelial shape and function are lost alongside ECM breakdown by the increased expression of matrix metalloproteinases [MMPs], including MMP9 [33]. The stage prior to immune cell infiltration is associated with acinar cell apoptosis and basal lamina destabilization due to specific proteolytic enzymes secreted by acinar and ductal cells, i.e., MMP9 [34,35]. Early MMP9 dysregulation and ECM destabilization of acini structure may occur before infiltration or distally from infiltrates, as observed in *sicca* patients and the nonobese diabetic/severe combined immunodeficiency [NOD-scid] mouse model of SjD, indicating that multiple pathways could be involved in SjD onset, possibly related to genetic and epigenetic changes [29,33,34,35]. Mechanisms driving MMP9 expression may shift during the early sicca to clinical SjD stages. ECM integrity loss could impair the feedback loop necessary for re-epithelization of damaged acinar epithelium by progenitor cells through EMT (Figure 2, Section 5) [36]. Ineffective restoration by resident progenitor cells causes acinar structures to remain in a constant state of disrepair. Exhaustion of progenitor cells might occur due to telomere-induced senescence, possibly exacerbated by lifetime exposure to reactive oxygen species [ROS] involving somatic epigenetic changes (Figure 2, Section 5) [37]. 

During the clinical phase, disorganization and disrepair of the salivary epithelium occur in acinar and ductal cell populations, exhibiting changes to aquaporin localization and/or expression, altered cell volume, and improper intracellular calcium signaling (Figure 2, Section 6a) [26]. This results in a loss of function of the secretory cells (Figure 2, Section 6b) [38]. The chronic state of dysfunctional repair within the salivary epithelium during SjD pathogenesis leads to substantial infiltration by immune cells into the glandular tissue, mainly within striated ducts, while CD8+ cells may accumulate within the acini, along with CD4+ T cells, macrophages, dendritic cells, and B cells (Figure 2, Section 7) [26].

### 3.2. Challenges in Targeting the Pre-Symptomatic Phase during SjD Development

The classification of SjD subsets and varying treatment efficacy suggest a more individual-targeted approach to mitigate SjD progression and manifestations [39]. The use of metrics outside of autoantibody profiling and the determination of lymphocytic infiltration (e.g., salivary flow rate or composition) could provide an alternative strategy for classifying disease progression [39]. A study by Theander et al. concluded that the presence of anti-SSA/SSB was associated with systemic manifestations and earlier disease onset [24]. These studies highlight the multifactorial and etiological nature of SjD, due to which characterizing SjD subsets by immunologic and non-immunologic measures would be helpful in identifying more efficacious treatments among distinct patient subsets. Indeed, about 10% of *sicca* patients may progress to SjD, and immunological factors were found to be indicative of such progression [40].

SjD development can vary depending on the age of onset and the presence of autoantibodies [24]. Predicting disease progression through longitudinal studies has been mostly elusive. To address the lack of *sicca* in SjD transitional biopsy samples, most early SjD pathogenesis has been elucidated through mouse models. Due to the usual late stage of SjD classification, treating *sicca* patients would likely involve targeting mechanisms that are not necessarily significantly controlled by the immune system. Focusing research on early changes to salivary glands and oral epithelia could provide candidate surrogate biomarkers predictive of disease progression towards chronic systemic autoimmunity in SjD.

Another challenge surrounding clinical trial design is the 7.5-year time lapse between symptom onset and SjD diagnosis [41]. In a case-control study by Pertovaara et al., roughly 36% of subclinical *sicca* patients were later diagnosed with SjD, whereas 50% of subjects with subclinical levels of lymphocytic infiltrates demonstrated an increase in sialadenitis after an 11-year (median time) follow-up biopsy [42]. Additionally, Pertovaara et al. demonstrated an increase in anti-SSA (40%), anti-Sjögren’s syndrome type B [anti-SSB] (33%), and antinuclear autoantibodies ([ANA]; 58%) levels from 5%, 4%, and 20%, respectively, after diagnosis of SjD compared to *sicca* patients at an 11-year (median time, range of 8–17 years) follow-up visit [42]. These findings differ slightly from those of Theander et al., 2015, who described autoantibody levels to range from 51 to 29% for anti-SSA/SSB and 68% for ANA during a median time of 4.3–5.1 years before SjD classification has been established [24]. Both studies indicated ANA prevalence after SjD classification to be greater than either anti-SSA or anti-SSB. However, ANA was detected at relatively high levels in both SjD and *sicca* groups compared to anti-SSA and anti-SSB antibodies when present [24,42]. Overall, studies by Pertovaara et al. and Theander et al. identified similar processes in SjD-related pathogenesis, possibly involving somatic genetic and epigenetic changes, years prior to symptom onset. 

Genetic and epigenetic modifications could significantly impact the molecular make-up of salivary epithelial cells governing SjD predisposition and the appearance of autoantibodies associated with the epithelial–immune cell interplay, thereby providing new opportunities for therapeutic development [26,35]. Such modifications could lead to the accumulation of damage-associated molecular patterns [DAMPs], disrupting the response to gamma-interferon [IFNγ] or the innate immunity response to dsDNA leaking from damaged cells and mitochondria, further exacerbated by interleukin-33 expression, leading to chronic and systemic inflammation [43].

Early treatment may thus aid in preserving salivary gland function rather than restoring salivary gland structural integrity. The need for early treatment is supported by the similarity between salivary gland expression profiles of *sicca* patients compared to SjD patients without a positive biopsy [44]. Transcriptomic levels of MLSG biopsies paired with parotid gland biopsies were similar among SjD and *sicca* patients [44].

## 4. Loss of Structural Integrity and Salivary Hypofunction of Acini Structures in SjD

### 4.1. Loss of ECM Integrity in SjD 

In SjD, extensive and chronic ECM degradation by MMPs (i.e., MMP9) can alter the salivary epithelium to an undifferentiated and disorganized state to a certain degree [32]. Indeed, the salivary epithelium relies on ECM integrity and progenitor cell maintenance/replication, as exemplified by the aberrant expression of the epithelial cellular adhesion molecule [EpCAM] in MLSGs, a factor regulating EMT [45]. Connective interactions between epithelial cells and acinus structure depend on ECM integrity. The ECM mediates cell migration, function, and differentiation through protein–protein interactions, as shown in fibrotic diseases and cancer [46]. The ECM’s main components are collagen (type I, IV), laminin, nidogen, proteoglycans, and fibronectin [46]. In salivary gland development, these components interact with trans-membrane protein integrin (cell–ECM connection, i.e., hemidesmosomes), providing major signaling cues for establishing cell polarity and, conversely, cell–cell interactions [47]. In SjD, these interactions involve tight and adherens junctions [48]. The ECM and cell–cell connections mediate the polarized secretion of acinar cells into the salivary lumen. 

**Figure 3:**
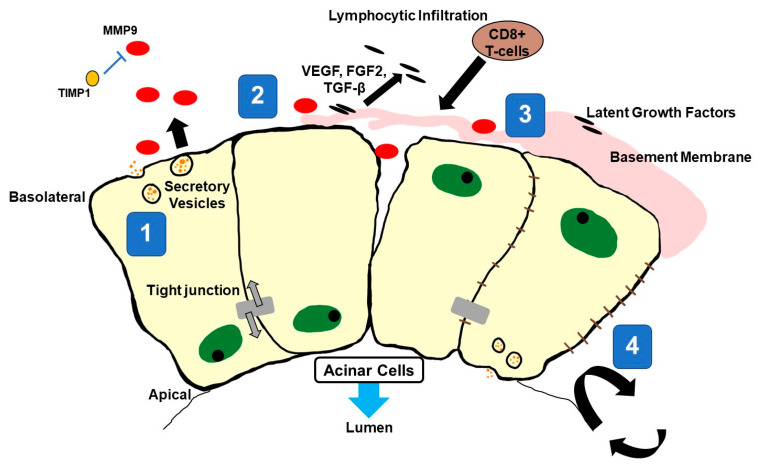
Potential disruptions in acinar cell function and homeostasis by MMP9 in SjD. Legend. Epithelial function and integrity are reliant upon both internal and external factors for homeostasis. External factors may include the integrity of cell–cell connections (tight junctions) and cell connections with the extracellular matrix [ECM]. Changes or loss of these external factors can disrupt epithelial homeostasis and initiate pathologic changes, which have been observed in the glandular epithelium of SjD patients. Disruption of both tight junctions and cell–ECM connections has been attributed to the overexpression of matrix metalloproteinase 9 [MMP9], which may be overexpressed or more active due to reduced levels of the TIMP metallopeptidase inhibitor 1 [TIMP1] in the salivary epithelium of both *sicca* and SjD patients. MMP9 can degrade multiple components within cell–cell junctions and the surrounding ECM. (1) Degradation of tight junctions by MMP9 can alter the directional secretory abilities of epithelial cells. (2) Loss of tight junctions causes cellular components located within apical/basolateral sections to diffuse among the plasma membrane, inhibiting directional (polarized) secretion. Tight junctions also facilitate paracrine-mediated signaling, which is subsequently compromised within the epithelium. (3) Breakdown of the ECM and tight junctions increases the permeability of the epithelium, in turn facilitating lymphocytic infiltration mostly by CD8+ T-cells into acini. ECM degradation products reciprocally act as chemo-attractants for lymphocytes, further driving tissue infiltration. Latent growth factors such as vascular endothelial growth factor [VEGF], fibroblast growth factor 2 [FGF2], and transforming growth factor beta 1 [TGF-β] sequestered within the ECM are released and proteolytically activated by MMP9. (4) After subsequent damage to tissues, re-epithelization of the damaged region by resident stem/progenitor cells relies on external cues from the surrounding ECM; however, MMP9 overexpression and excessive breakdown of ECM structures could impair healing and repair of the glandular epithelium in SjD patients.

Beyond cell polarity, the ECM directs epithelial cell differentiation and developmental signals [48]. ECM components provide signaling responses to the epithelia in a feedback loop, driving either ECM degradation through EMT or epithelial differentiation. ECM degradation products inhibit epithelial cell differentiation (e.g., transforming growth factor beta 1 [TGFβ1], collagen IV) and lead to the expression of EMT-associated factors (i.e., snail family genes, zinc finger E-box binding homeobox ½ gene) [49,50]. In SjD, upon significant EMT changes, MMP9 overexpression or increased activity due to reduced negative regulation by TIMP metallopeptidase inhibitor 1 [TIMP1] may impair salivary gland acinar cell function [33]. This is presented in Figure 3, focusing on acinar structure pathology. 

Epithelial cell–ECM interactions not only mediate epithelial homeostasis but are essential during salivary gland development [32,47]. Integrin receptors control signaling pathways critical to cell function and are major sources of external signals for differentiation [51]. Integrins are commonly located in the plasma membrane, where they act as receptors for various external constituents primarily located within the innermost layer of the ECM, the basement membrane (e.g., laminin) [52]. The basement membrane is responsible for signaling through integrin receptors both the orientation and survival of the epithelium, alongside several other functions [51]. For instance, integrin alpha 6 beta 4 [α6β4] has been shown to control the expression of genes linked to apoptosis [52]. Signaling by α6β4 can lead to the demethylation of select promoters [52]. In SjD patients, α6β4 has been linked to acinar cell death in cases where there is severe basal lamina disorganization [53]. 

Tight and adheren junctions (cell–cell) are essential for preserving the barrier function of epithelial (acinar) cells and maintaining their ability for polarized secretion alongside hemidesmosomes (ECM–cell) [48]. Ultimately, acinar cell depolarization alters tight junctions and the expression of e-cadherin (Cadherin 1 [CDH1]) [54,55,56]. This process may cause acinar cells to detach and adopt a mesenchymal phenotype, as part of the EMT process in salivary and lacrimal glands [49]. Excessive degradation of extracellular structures may thus affect both epithelial cell function (polarity, secretion) and repair involving progenitor cell duplication, re-epithelization, and EMT in SjD development. As shown in Figure 3, lymphocytes infiltrating acinar structures are mainly CD8+ T cells [4].

### 4.2. Aberrant Expression of EMT-Associated Genes in the Salivary Epithelium of SjD

Glandular formation and repair are mediated through EMT and epithelial–mesenchymal plasticity [EMP] processes. Feedback responses from the ECM dictate epithelial migration, secretion (i.e., new basement membrane), and structure formation through multiple forms of EMT processes (Type I, II, III) [57]. Type I EMT is associated with embryo formation from the blastula to the gastrula stage [57]. Type II EMT is most relevant to SjD pathology and is associated with wound healing consisting of hemostasis, inflammation, proliferation, and remodeling biological processes [58]. Type III EMT has been associated with the mesenchymal shift of epithelial cancer cells and metastasis [57]. Moreover, Type II EMT is associated with fibrosis occurring during advanced SjD stages [59]. Cells undergoing Type II EMT express several epithelial markers such as cytokeratin and CDH1, while simultaneously expressing markers with mesenchymal phenotypes such as *alpha*-smooth muscle actin [α-SMA] and vimentin [57,60]. In recent years, EMP has been related to intermediary stages of EMT, where cells exhibit both epithelial and mesenchymal expression markers, but has not yet been fully investigated in SjD [5]. 

### 4.3. Dysregulation of ETS1 and MMP9 Expression in SjD Salivary Glands

ETS Proto-Oncogene 1 [ETS1] belongs to the ETS family of transcription factors that bind to a conserved (GGAA/T) DNA sequence. Pathways involving glandular morphogenesis, tissue remodeling, EMT, leukocyte migration, differentiation, and cytokine/chemokine expression have been linked to ETS1 expression [57]. One important concept is the significance of ETS1 in MMP9 expression and cancer metastasis [61]. ETS1 upregulates MMP9 through transcription control by directly binding the MMP9 promoter [62]. During glandular morphogenesis and cancer metastasis, involving EMT-driven processes, ETS1 participates by modulating ECM–cell interactions (i.e., adhesion, integrins, MMPs, collagen) and EMT transcription factor expression [63,64]. Moreover, ETS1 is associated with defects in the B-cell maturation of SLE and the alteration of epithelial responses to chemokines and cytokines [65]. However, the involvement of ETS1 in SjD and salivary gland homeostasis requires further characterization.

Extracellular matrix remodeling is a significant intermediary process during EMT [57]. Dysregulated factors within the salivary epithelium of salivary gland tissue biopsies or primary cultures of the SGECs of SjD patients are shown in Table 1. In addition to the dysregulated or chronic dysregulated EMT/EMP response involving these factors, we showed the overexpression of ETS1 alongside MMP9 in SGECs of SjD patients without the proximity of infiltrating CD4+ lymphocytes [29]. ETS1 has been previously shown to regulate MMP9 expression in multiple epithelial cancer cell models [66]. ETS1 and MMP9 can regulate EMT and have both been frequently associated with cancer metastasis [63,67]. 

## 5. Conclusions and Future Directions

Current diagnostic markers for SjD are fundamentally aimed at immune involvement, with significant limitations to associating disease activity with the rate of progression. Biomarkers indicative of the pathogenesis involving the salivary epithelium could help in assessing SjD progression or treatment response. The salivary glands of SjD patients display a disorganized EMT process, contributing to the hypofunction of the salivary gland epithelia. 

With further knowledge of underlying etiological factors and molecular mechanisms impacting early disease processes, therapeutic targets could be identified to prevent unaffected salivary gland tissue damage by substantial lymphocytic infiltration in *sicca* patients. Factors affecting ETS1 expression, or factors affected by ETS1 prior to symptom manifestation, may be monitored alongside the presence of serum autoantibodies. Targeting the initial production of MMP9 through ETS1, using local delivery systems, would thus provide a viable therapeutic strategy for combating glandular structural deterioration and salivary hypofunction in SjD. Overall, reducing early overexpression/increased activity of MMP9 could preserve glandular architecture, thereby limiting lymphocytic infiltrates and the loss of structural integrity of the acinar structure. Accordingly, simultaneous RNA and protein expression analysis at the single-cell level will likely provide valuable insights into the spatial changes that acinar and ductal cells undergo in relation to the development of autoimmunity since the loss of structural and functional integrity is heterogeneous across salivary gland tissues over time.

## Figures and Tables

**Table 1:** EMT-associated factors dysregulated in the salivary epithelium or cultured SGECs of SjD patients.

Gene/Protein	Increase/Decrease or Altered Localization	Potential Significance	PMID/s
ETS1	Increase	Upregulates MMP9 through transcription control by directly binding the MMP9 promoter.	21862874; 28818099; 31165469; 36008454
LEF1	Increase	Transcription factor that regulates EMT.	21862874; 28818099; 31165469
MMP9	Increase	Enhanced expression/activity can damage ECM. Early MMP9 dysregulation may occur before immune cell infiltration.	16142742; 17689946; 23497939; 28421997; 28818099; 31165469; 36008454
Vimentin	Increase	Shown to be upregulated in SjD and may be an indicator of the extent of inflammation.	21862874; 29789993; 31165469
TGF- β1	Increase	Proteolytically activated by MMP9.	21862874; 29789993
SMAD4	Increase	Involves TGF-β and receptors to activate EMT leading to the activation of RAS/RAF/MEK/ERK/MAPK pathways.	29789993
Type-1 Collagen	Increase	Type-1 collagen is degraded with increased degradation of lacrimal gland ECM structures, implicating a key event in SjD development.	17689946; 19011242; 29789993; 31165469
SNAI1	Increase	Factor associated with EMT expression due to inhibited epithelial cell differentiation.	19011242; 21862874; 29789993
Claudin-1	Increase, Altered Localization	Higher protein levels in salivary gland tight junctions in patients with SjD.	20131287; 21862874; 23160379
Claudin-3	Altered Localization	Shown to be redistributed to basolateral plasma membrane of SjD patients.	20131287; 21862874
Claudin-4	Increase, Altered Localization	Higher protein levels in salivary gland tight junctions in patients with SjD.	20131287; 21862874
E-cadherin/CDH1	Decrease	May cause acinar cells to detach and adopt a mesenchymal phenotype.	21862874; 29789993; 31165469
Occludin	Decrease, Altered Localization	Apical domain presence of occludin is decreased in patients with SjD.	20131287
ZO1	Decrease, Altered Localization	Apical domain presence of ZO1 is decreased in patients with SjD.	20131287
α6β4 integrin	Altered Localization	Detected in the cytoplasm and lateral plasma membrane in serous and mucous acini showing dramatic alterations in acini with strong basal lamina disorganization of patients with SjD.	18625620; 19011242
ZEB2	Increase	Shown to be upregulated during the initiation and progression of multiple EMT subtypes.	21862874; 36759947
SLUG	Decrease	Shown to be downregulated in SGEC’s of SjD patients compared to controls.	36759947

Factors previously linked to or demonstrated to regulate EMT and found to be dysregulated within the salivary epithelium or cultured SGECs of SjD patients are shown.

## Supplementary Materials

The following supporting information can be downloaded at https://www.mdpi.com/article/10.3390/ijms25094973/s1.
