# Supplementary material for: Exploring Salivary Epithelial Dysfunction in Sjögren’s Disease
Source: Int J Mol Sci. 2024 May 2;25(9):4973. doi: 10.3390/ijms25094973 (PMC11084897; doi:10.3390/ijms25094973)
Supplement: Supplementary file 1 [file ijms-25-04973-s001.zip › Supplemental File S2. SjD review risk of bias Tables 04 19 2024 FINAL.pdf]

**Supplemental File S2**. Risk of bias assessment per PRISMA guidelines

**a.** Main manuscript references

Risk of bias domains

| Study | D1 | D2 | D3 | D4 | D5 | Overall |
|---|---|---|---|---|---|---|
| Molina et al., 2006 | + | - | + | + | + | - |
| Gilboe et al., 2001 | - | - | + | + | + | - |
| Shiboski et al., 2017 | + | + | + | + | + | + |
| Radfar et al., 2002 | - | + | + | + | + | - |
| Garreto et al., 2021 | + | + | + | + | + | + |
| Theander et al., 2015 | + | + | + | + | + | + |
| Verstappen et al., 2021 | + | + | + | + | + | + |
| Seror et al., 2015 | + | + | + | + | + | + |
| Shah et al., 2019 | + | + | + | + | + | + |
| Fujita-Yoshigaki et al., 2008 | + | + | + | + | + | + |
| Perez et al., 2005 | + | + | + | + | + | + |
| Robinson et al., 1996 | - | + | + | + | + | - |
| Scott et al., 2019 | + | + | + | + | + | + |
| Kawashima et al., 2011 | - | + | + | + | + | - |
| Bharaj et al., 2021 | + | + | + | + | + | + |
| Shiboski et al., 2018 | + | + | + | + | + | + |
| Sebastian et al., 2018 | + | + | + | + | + | + |
| Pertovaara et al., 1999 | + | + | + | - | - | - |
| Soyfoo et al., 2021 | + | + | + | + | + | + |
| Zhang et al., 2019a | - | + | + | - | + | - |
| Cox et al., 2011 | + | + | + | + | + | + |
| Wu et al., 2019 | - | + | + | + | + | - |
| Chen et al., 2009 | + | + | + | + | + | + |
| Velozo et al., 2009 | - | + | + | + | + | - |
| Zhang et al., 2019b | + | + | + | + | + | + |
| Leehan et al., 2017 | + | + | + | + | + | + |
| Jordan et al., 2011 | + | + | + | + | + | + |
| Furlan et al., 2008 | + | + | + | + | + | + |
| Noll et al., 2022 | - | + | + | + | + | - |
| Wang et al., 2005 | - | + | + | + | + | - |

Domains:
D1: Bias arising from the randomization process.
D2: Bias due to deviations from intended intervention.
D3: Bias due to missing outcome data.
D4: Bias in measurement of the outcome.
D5: Bias in selection of the reported result.

Judgement
- Some concerns
+ Low

**b.** Figure 1 and Table 1 references

Risk of bias domains

| Study | D1 | D2 | D3 | D4 | D5 | Overall |
|---|---|---|---|---|---|---|
| Wise and Woodruff, 1993 | + | + | + | + | - | - |
| Fayyaz et al., 2016 | + | + | + | + | + | + |
| Radfar et al., 2002 | - | + | + | + | + | - |
| Daniels et al., 2011 | + | + | + | + | + | + |
| Risselada et al., 2013 | + | + | + | + | + | + |
| Risselada et al., 2014 | + | + | + | + | + | + |
| Carubbi et al., 2015 | + | + | + | + | + | + |
| Wei et al., 2015 | + | + | + | + | + | + |
| Theander et al., 2015 | + | + | + | + | + | + |
| Leehan et al., 2018 | + | + | + | + | + | + |
| Kakugawa et al., 2018 | + | + | + | + | + | + |
| Abd-Allah et al., 2019 | + | + | + | + | + | + |
| Jordan et al., 2011 | + | + | + | + | + | + |
| Shah et al., 2017 | + | + | + | + | + | + |
| Shah et al., 2019 | + | + | + | + | + | + |
| Noll et al., 2022 | - | + | + | + | + | - |
| Perez et al., 2005 | + | + | + | + | + | + |
| Schenke-Layland et al., 2008 | + | + | + | + | + | + |
| Barrera et al., 2013 | + | + | + | + | + | + |
| Tandon et al., 2017 | - | + | + | + | + | - |
| Sisto et al., 2018 | + | + | + | + | + | + |
| Sisto et al., 2019 | + | + | + | + | + | + |
| Chen et al., 2010 | + | + | + | + | + | + |
| Ewert et al., 2010 | + | + | + | + | + | + |
| Velozo et al., 2009 | - | + | + | + | + | - |
| Riviere et al., 2023 | + | + | + | + | + | + |

Domains:
D1: Bias arising from the randomization process.
D2: Bias due to deviations from intended intervention.
D3: Bias due to missing outcome data.
D4: Bias in measurement of the outcome.
D5: Bias in selection of the reported result.

Judgement
- Some concerns
+ Low

**Footnote:**

Risk-of-Bias assessment table using 'Cochran's Handbook for Systematic Reviews of Interventions' Risk-of-bias VISualization (robvis) tool for **a.** references in the main manuscript excluding reviews and **b.** references from Figure 1 and Table 1 manually curated using conventional searches of PubMed and PubMed Central for articles related to Sjogren's Disease in English.
